# Surveillance of Hepatocellular Carcinoma in Nonalcoholic Fatty Liver Disease

**DOI:** 10.3390/diagnostics10080579

**Published:** 2020-08-10

**Authors:** Yoshio Sumida, Masashi Yoneda, Yuya Seko, Hiroshi Ishiba, Tasuku Hara, Hidenori Toyoda, Satoshi Yasuda, Takashi Kumada, Hideki Hayashi, Takashi Kobayashi, Kento Imajo, Masato Yoneda, Toshifumi Tada, Takumi Kawaguchi, Yuichiro Eguchi, Satoshi Oeda, Hirokazu Takahashi, Eiichi Tomita, Takeshi Okanoue, Atsushi Nakajima, Japan Study Group of NAFLD (JSG-NAFLD)

**Affiliations:** 1 Division of Hepatology and Pancreatology, Department of Internal Medicine, Aichi Medical University, Nagakute, Aichi 480-1195, Japan; yoneda@aichi-med-u.ac.jp; 2 Department of Gastroenterology and Hepatology, Graduate School of Medicine, Kyoto Prefectural University of Medicine, Kyoto 602-8566, Japan; yuyaseko@koto.kpu-m.ac.jp; 3 Department of Gastroenterology, Japanese Redcross Kyoto daiichi Hospital, Kyoto 605-0981, Japan; chiroinu@koto.kpu-m.ac.jp; 4 Department of Gastroenterology, Fukuchiyama City Hospital, Fukuchiyama, Kyoto 620-8505, Japan; t-hara@koto.kpu-m.ac.jp; 5 Department of Gastroenterology, Ogaki Municipal Hospital, Ogaki, Gifu 503-8502, Japan; hmtoyoda@spice.ocn.ne.jp (H.T.); satoshi.yasuda.1982@gmail.com (S.Y.); takashi.kumada@gmail.com (T.K.); 6 Department of Gastroenterology, Gifu Municipal Hospital, Gifu 500-8513, Japan; hide-hayashi@umin.ac.jp (H.H.); etomita_jp@yahoo.co.jp (E.T.); 7 Department of Gastroenterology and Hepatology, Yokohama City University Graduate School of Medicine, Yohokama, Kanagawa 236-0004, Japan; takobayashi-hok@umin.ac.jp (T.K.); kento318@yokohama-cu.ac.jp (K.I.); yoneda-ycu@umin.ac.jp (M.Y.); nakajima-tky@umin.ac.jp (A.N.); 8 Department of Hepatology, Himeji Redcross Hospital, Himeji, Hyogo 670-8540, Japan; tadat0627@gmail.com; 9 Division of Gastroenterology, Department of Medicine, Kurume University School of Medicine, Kurume 830-0011, Japan; takumi@med.kurume-u.ac.jp; 10 Loco Medical General Institute, 1178-1 Kanada Mikatsuki Ogi, Saga 849-8501, Japan; eguchiyu@eguchi-hospital.com; 11 Liver Center, Saga Medical Hospital, Saga, Saga 849-8501, Japan; takahas2@cc.saga-u.ac.jp (H.T.); ooedasa@edu.cc.saga-u.ac.jp (S.O.); 12 Hepatology Center, Saiseikai Suita Hospital, Suita, Osaka 564-0013, Japan; okanoue@suita.saiseikai.or.jp; 13 Japan Strategic Medical Administration Research Center (J-SMARC), Nagoya, Aichi 460-0011, Japan

**Keywords:** hepatic fibrosis, Mac-2 binding protein glycated isomer, apoptosis inhibitor of macrophage, patatin-like phospholipase domain-containing protein 3, α-fetoprotein, protein induced by vitamin K absence or antagonist-II

## Abstract

Nonalcoholic fatty liver disease (NAFLD) is becoming the leading cause of hepatocellular carcinoma (HCC), liver-related mortality, and liver transplantation. There is sufficient epidemiological cohort data to recommend the surveillance of patients with NAFLD based upon the incidence of HCC. The American Gastroenterology Association (AGA) expert review published in 2020 recommends that NAFLD patients with cirrhosis or advanced fibrosis estimated by non-invasive tests (NITs) consider HCC surveillance. NITs include the fibrosis-4 (FIB-4) index, the enhanced liver fibrosis (ELF) test, FibroScan, and MR elastography. The recommended surveillance modality is abdominal ultrasound (US), which is cost effective and noninvasive with good sensitivity. However, US is limited in obese patients and those with NAFLD. In NAFLD patients with a high likelihood of having an inadequate US, or if an US is attempted but inadequate, CT or MRI may be utilized. The GALAD score, consisting of age, gender, AFP, the lens culinaris-agglutinin-reactive fraction of AFP (AFP-L3), and the protein induced by the absence of vitamin K or antagonist-II (PIVKA-II), can help identify a high risk of HCC in NAFLD patients. Innovative parameters, including a Mac-2 binding protein glycated isomer, type IV collagen 7S, free apoptosis inhibitor of the macrophage, and a combination of single nucleoside polymorphisms, are expected to be established. Considering the large size of the NAFLD population, optimal screening tests must meet several criteria, including high sensitivity, cost effectiveness, and availability.

## 1. Introduction

**Figure 1:**
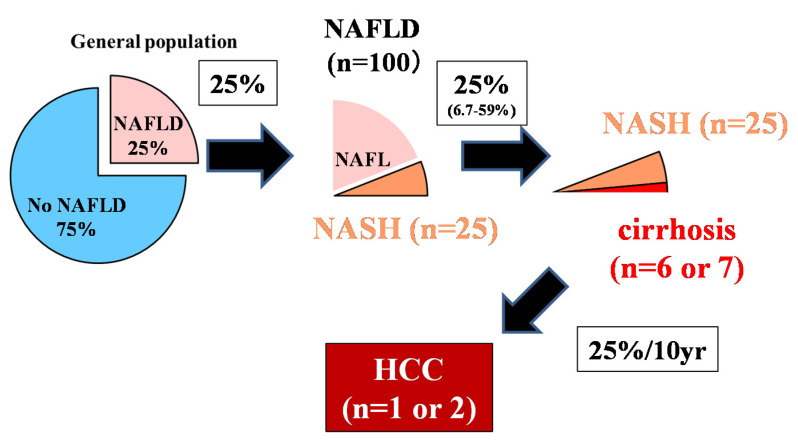
The 25% rule in nonalcoholic fatty liver disease (NAFLD) [10].

The control of viral hepatitis (hepatitis B virus (HBV) and hepatitis C virus (HCV)) has now become possible, and the so-called non-HBV non-HCV hepatocellular carcinoma (NBNC-HCC) has become 1/3 of the total number of HCC cases in Japan [1]. The main background of NBNC-HCC is fatty liver disease (FLD), which is caused by alcohol consumption and/or lifestyle-related factors [1,2]. In the past, low-drinking FLD was called nonalcoholic fatty liver disease (NAFLD). It has been proposed to change this name to metabolic dysfunction associated fatty liver disease (MAFLD) [3]. NAFLD comprises a broad spectrum of syndromes, ranging from simple steatosis and nonalcoholic steatohepatitis (NASH) to fibrosis, cirrhosis, and HCC [4]. Some NAFLD patients with the progression of fibrosis experience liver disease-related mortality (HCC, liver failure, or esophageal varices hemorrhaging) or require liver transplantation [5]. NAFLD affects about 25% of adults [6,7], but about 25% (6.7%–59%) transition to NASH [8], 25% of whom develop cirrhosis. Since 25% of cancers occur over 10 years [9] (Figure 1, 25% rule), it is estimated that only 1 or 2 out of every 100 NAFLD cases develop HCC (Figure 1) [10,11]. Although it is clear that NAFLD portends a lower risk for HCC than HBV or HCV, the high prevalence of NAFLD in the population underlies the importance of NAFLD in the development of HCC [12]. However, poor surveillance is a constant problem for patients with NAFLD. According to cohort studies from Italy and the United States, many patients with NAFLD-related HCC are not diagnosed through regular surveillance (compared to patients with HCV-related HCC), resulting in a more advanced HCC burden at diagnosis [13,14]. This review outlines the most efficient types of surveillance for HCC in NAFLD.

Although 25% of adults have NAFLD, about 25% will progress to NASH in their lifetime, and 25% will progress from NASH to liver cirrhosis. The incident rate of HCC 10 years after liver cirrhosis is about 25%. Out of 100 NAFLD patients, it is rare for 1–2 people with NAFLD to develop HCC. The authors have copyright of this figure [10].

## 2. Carcinogenic Risk in Nonalcoholic Fatty Liver Disease

The risk of hepatocarcinogenesis from NAFLD varies depending on the background of the population. Globally, HCC incidence among NAFLD patients is 0.44 per 1000 person-years (range, 0.29–0.66) [6]. Comparing 296,707 NAFLD patients with 296,707 matched controls without known liver disease in the United States National Veterans Health Administration system, the incident HCC rate was shown to be 0.02 per 1000 person-years in normal subjects and 0.21 per 1000 person-years in NAFLD. Those with NAFLD have a higher risk compared to healthy people (hazard ratio [HR] 7.62, 95% confidence interval [CI] 5.76–10.09) [15]. In a study comparing the incidence of HCC among patients with HCV infection and NAFLD [16], 315 patients with HCV-cirrhosis and 195 with cirrhosis due to NAFLD were followed for a median of 3.2 years. The cumulative incidence of HCC was slightly lower for NAFLD-related cirrhosis than for HCV cirrhosis (2.6% vs. 4%, *p* = 0.09) [16]. In a Danish cohort study, none of the 170 subjects in the NAFLD cohort without significant fibrosis at baseline developed HCC during an average of almost 21 years follow-up [17]. A Swedish cohort study over 21 years found 3% and 6% cumulative HCC mortality for biopsy-proven NAFLD and NASH, respectively [18]. In Japan, the annual rate is 0.04% for cases of NAFLD diagnosed by ultrasonography (US) [19], 0.4%–0.8% for cases of NAFLD diagnosed by liver biopsy [20], and 2% to 3% for those with NASH associated cirrhosis [9]. In this way, the HCC risk in NAFLD seems to be limited to individuals with cirrhosis [21].

The best available evidence suggests that NAFLD-related cirrhosis is a risk factor for HCC, albeit at a lower rate compared to HCV-related cirrhosis. However, the annual incidence rate of NASH-cirrhosis remains higher than 1%. HCC has also been observed in NAFLD patients without cirrhosis but with incidence rates lower than 1% a year [22,23]. The surveillance of HCC in every patient with NAFLD is unrealistic. However, screening for HCC in cirrhotic patients is justifiable based on financial considerations. An important issue is how to determine high-risk cases from a large number of NAFLD patients for early diagnosis and treatment of HCC. Advanced fibrosis (F3/4), old age, male gender, low platelets (less than 150,000/μL), high AST, diabetes, and the patatin-like phospholipase domain-containing protein 3 (PNPLA3) single nucleotide polymorphism (SNP) GG homozygote have been established as carcinogenic risk factors in Japan [9,19,21]. These results are consistent with the data from other Asian and western countries [15,16,24,25,26]. We should also clarify the risk factors for HCC incidence in NASH-cirrhosis patients. Among 354 patients with a diagnosis of NASH cirrhosis at the Mayo Clinic, Rochester, diabetes (HR = 4.2; 95% CI = 1.2–14.2; *p* = 0.02), age (per decade, HR = 1.8; 95% CI = 1.2–2.6; *p* < 0.01), and low serum albumin (HR = 2.1; 95% CI = 1.5–2.9; *p* < 0.01) were significantly associated with an increased risk of developing HCC in a multivariable analysis. Other metabolic risk factors, including body mass index (BMI), hyperlipidemia, and hypertension, were not associated with HCC risk [27].

## 3. Non-Invasive Diagnostic Method for Liver Fibrosis

The degree of liver fibrosis also contributes to the prognosis of NAFLD [25,28]. In the United States, HCC surveillance targets highly fibrotic cases (particularly liver cirrhosis) [29]. The American Association for the study of Liver Disease (AASLD) Practice Guide 2018 recommends four noninvasive tests (NITs) to evaluate hepatic fibrosis, including the Fibrosis-4 (FIB-4) index, the NAFLD fibrosis score (NFS) (Table 1), vibration-controlled transient elastography (VCTE), and magnetic resonance elastography (MRE) [30]. Kanwal et al. [15] showed that an FIB-4 index > 2.67 is associated with an increased risk of HCC not only in those with known cirrhosis but also in those without a prior diagnosis of cirrhosis. When utilizing NITs to risk stratify patients for HCC screening, a higher cutoff threshold is desirable to maximize specificity (90%). The following cutoff points for VCTE and MRE can be considered for the noninvasive detection of cirrhosis for the purposes of HCC screening: VCTE of 16.1 kPa and MRE of 5 kPa [29]. In recent years, a two-step diagnostic algorithm that combines these factors has become widespread [31,32,33] for stratifying patients with advanced fibrosis. The simple FIB-4 index is the first step, and the use of VCTE (FibroScan) is recommended mainly in the United States and Canada as the second step [34,35]. Since the FIB-4 index has a high negative predictive value, it is useful for excluding highly fibrotic cases. The FIB-4 index can be accurately used as the first step assuming the agreement of a primary care physician or health checkup facility. However, among hepatologists, the lowest cutoff value should be 1.45 [33,36], 1.3 [37], or 2.0 because the FIB-4 index can overpredict advanced fibrosis in the elderly [38,39]. That the FIB-4 index may show a false low value in diabetic patients [40] is not controversial, but this index remains sufficient for the first-step targeting of 2 billion NAFLD patients [31]. On the other hand, VCTE (FibroScan) is not widely used, and there are great expectations for serum markers. In Europe, the ELF (enhanced liver fibrosis) test, involving hyaluronic acid and a tissue inhibitor of matrix metalloproteinase type 1 (TIMP-1) along with P3NP (aminoterminal propeptide of type 3 procollagen) is used as the second step [41] (Table 1). A validation study for the efficacy of ELF test was conducted in Japan [42]. In Japan, liver fibrosis markers such as type IV collagen 7S and Mac-2 binding protein glycosylation isomer (M2BPGi) are generally used by hepatologists. Japanese patients with elevated type IV collagen 7s levels, reflecting severe fibrosis [43,44], are at increased risk of extrahepatic cancer and overall mortality, with biopsy-proven NAFLD [45]. Type IV collagen 7S was previously measured by the radioimmunoassay (RIA) method, but since August 2020, it has become possible to measure this marker via the high-sensitivity ELISA method. Future discussions should determine which parameter is best to use, but it is necessary to discuss not only diagnostic accuracy but also the cost–benefit balance, including medical economic efficiency [26,31].

## 4. HCC Surveillance in NAFLD Advocated by the American Gastroenterology Association

In 2020, eight recommendations (best practices) were published by the American Gastroenterology Association (AGA) for HCC surveillance in NAFLD patients [29] (Table 2). It is recommended that HCC surveillance be performed in cases of cirrhosis or in cases where NITs indicate severe liver fibrosis (Recommendations 1 and 2). According to the data of NASH-associated HCC from the Ministry of Health, Labor, and Welfare NASH research group (Director: Dr. Takeshi Okanoue, Saiseikai Suita Hospital, Osaka, Japan), most women (70%) develop HCC from cirrhosis, while men (70%) develop HCC without cirrhosis [46]. In Italy, Piscaglia et al. reported that 46.2% of NAFLD associated HCC cases occur without cirrhosis [13]. Similar results were reported by a German study, in which 41.7% of the cases did not feature cirrhosis [47]. It has also been reported that NASH entails a high risk of carcinogenesis from non-cirrhotic livers compared to other liver diseases in the United States [48]. However, the incidence of HCC in those with NAFLD and earlier stages of fibrosis (F0–F2) is extremely low and not precisely defined. The threshold incidence for the efficacy of surveillance (>0.25 life-years gained) is 1.5% per year [49], but NAFLD without cirrhosis relates to an annual incidence of HCC < 1.5% per year. Therefore, systematic HCC screening may not be prudent [15,49]. Although there is a higher risk of developing HCC among those with earlier stages of NAFLD than in those without NAFLD, the incidence rates and determinants of risk have not been well-quantified and are likely too low to justify routine screening at this point. The AASLD practice guide 2018 [12] suggests that the risk of HCC is significantly lower in those with NAFLD with no cirrhosis than in those with cirrhosis; surveillance is not recommended for these patients. The risk factors of carcinogenesis from non-cirrhotic NAFLD includes male gender, low alcohol consumption, and a high FIB-4 index [50]. Given the large number of cases with mild liver fibrosis, routine surveillance is irrational. However, it may be useful to focus on males, those who engage in light alcohol consumption, and those with a high FIB-4 index. Although AFP measurements are considered a tumor marker in the recommendations (Proposal 5), PIVKA-II had a higher positive rate than AFP in the data of the NASH research group of the Ministry of Health, Labor, and Welfare [46] and in the data in the Japan Study Group of NAFLD (JSG-NAFLD) from a multi-center Japanese study [51]. PIVKA-II may be superior to AFP for detecting NASH-HCC, although this possibility needs to be validated in an international study. In HCV-infected patients, HCC screening using biannual AFP, annual abdominal US, or triple phase computed tomography (CT) is more cost effective than engaging in no surveillance, with a cost effectiveness ratio less than $ 50,000 quality-adjusted life years (QALY) [52,53]. The AASLD guidance 2018 for HCC surveillance recommends surveillance using US with or without AFP every 6 months [12]. US is an inexpensive and noninvasive surveillance method without any risk of radiation exposure for the patient [12]. The AGA expert review recommends to consistently record the adequacy of the liver via US, including parenchyma heterogeneity, visualization of the entire liver, and beam attenuation because abdominal US results are often difficult to visualize in many cases of severe obesity. The visualized US score for HCC screening is graded into the following categories: A—no or minimal limitations; B—moderate limitations, as the examination may obscure small masses; and C—severe limitations, as the examination may miss focal liver lesions [29]. Consequently, if the US quality is inadequate (i.e., category C or, in some cases, category B), we recommend considering other imaging modalities (e.g., CT scans or magnetic resonance imaging [MRI]) for HCC screening (Proposal 5). Compared with multidetector CT (MDCT) and extracellular contrast media-enhanced MRI (ECCM-MRI), Gd-EOB-DTPA-MRI can be used as the first-choice imaging modality for the medical care of HCC among patients with hepatitis or liver cirrhosis in Japan [54], China [55], Thailand, and Korea [56]. The optimal intervals of imaging studies remain obscure. In the aforementioned meta-analysis by Singal et al. [57], US surveillance every 6 months significantly improved the sensitivity of detection for early stage HCC compared to annual exams. More frequent imaging (every 3 months) did not improve survival or increase the detection of small HCC lesions and is, therefore, not recommended [58]. It is also necessary to discuss the domestic best practices for this recommendation in NAFLD.

## 5. Novel Indicators for Predicting Incident HCC Risk

**Figure 2:**
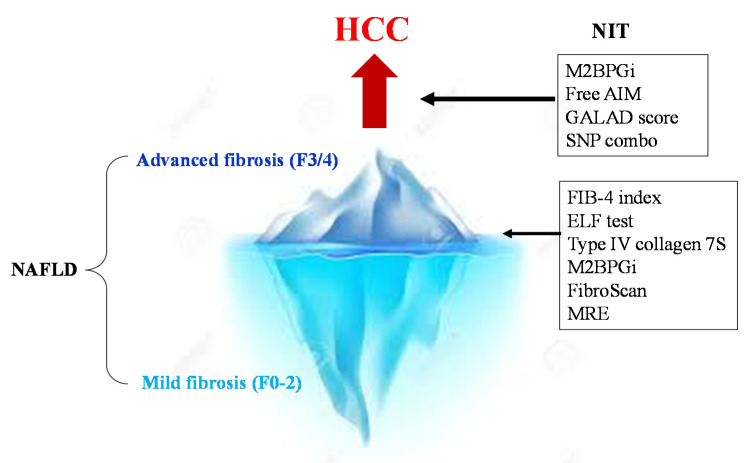
NITs for the surveillance of severe fibrosis and HCC in NAFLD. First, the NIT is used to determine advanced fibrosis (F3/4) from NAFLD. In cases of advanced fibrosis, regular image examinations are performed to detect HCC early. Then, strict surveillance is conducted among patients with a high GALAD score (>−0.63), high M2bpGi cases (>1.26), and PNPLA3 GG homozygous cases.

Methods for assessing the risk of hepatocarcinogenesis itself, rather than advanced fibrosis, have also been studied (Figure 2). The FIB-4 index and NFS are also useful for predicting cancer risk [59] (Table 1). In a national multicenter study led by JSG-NAFLD, Kawaguchi et al. reported that a favorable prognostic factor in NASH-HCC is serum albumin of 4.0 g/dL or more; further, the early detection of HCC can provide an indication of curative treatments, such as surgery and radiofrequency ablation therapy [60]. This suggests the importance of diagnosing HCC at an early stage when the hepatic reserve is maintained. Most NASH-HCC patients do not undergo regular surveillance. Consequently, their tumor sizes are large at the time of diagnosis, resulting in a poor prognosis [61,62].

### 5.1. Mac2 Protein Glycosylated Isomer

Mac-2-binding protein (M2BP) is a secretory glycoprotein that contains seven *N*-glycans per monomer [63]. In the serum, 10–16 monomers of M2BP form a doughnut-shaped polymer that presents 70–112 *N*-glycans. Alterations in M2BP occur during the progression of liver disease and fibrosis due to changes in N-glycosylation (i.e., sialylation or the extension of polylactosamine). However, the underlying mechanism is unclear. As a robust lectin that binds the GalNAc residue of *N*-glycans and *O*-glycans and the clustered LacNAc structure, Wisteria floribunda agglutinin (WFA) can recognize the altered *N*-glycans of M2BP specifically. Thus, this specific glycoprotein was described as WFA^+^-M2BP and renamed to M2BPGi after the commercialization of the diagnostic reagent. Level of WFA^+^ -M2BP in the sera were measured by a HISCL™ M2BPGi™ assay kit using an automated immunoanalyzer (HISCL™-800; Sysmex, Kobe, Japan). The measured values of WFA^+^ -M2BP conjugated to WFA were indexed with the obtained values using the following equation: Cutoff index (C.O.I) = ([WFA^+^-M2BP]sample-[WFA^+^-M2BP]_NC_)/([WFA^+^-M2BP]_PC_ − [WFA^+^ -M2BP]_NC_), where the [WFA^+^-M2BP] sample is the WFA^+^-M2BP count of the serum samples, PC is positive control, and NC is the negative control. The positive control was supplied as a calibration solution preliminarily standardized to yield a cutoff value of 1.0 [64]. The clinical application of the Wisteria floribunda agglutinin-positive Mac-2-binding protein (WFA^+^-M2BP) has been widely promoted after Japanese public health insurance began to cover its expenses in 2015. M2BPGi has been used as a liver fibrosis marker for various liver diseases [65], including NAFLD [43,64,66]. Accumulating evidence suggests that higher levels of serum M2BPGi can predict HCC incidence in patients with chronic hepatitis B [67,68,69,70,71,72]. According to a report by Kawanaka et al. [73], the carcinogenic rate was as high as 6.8% at 5 years and 21.1% at 10 years in NAFLD cases where M2BPGi was 1.26 or higher, while the rate was as low as 1.7% at 5 years and 1.7% at 10 years in patients with M2BPGi below 1.26 [73]. It has been suggested that M2BPGi may be a predictor of hepatocarcinogenesis, as well as fibrosis, but its mechanism has not been clarified.

### 5.2. GALAD Score

In Japan, AFP, the AFP-L3 fraction, and PIVKA-II (des-γ-carboxy pro-thrombin [DCP] in foreign countries) have been used for many years as tumor markers. According to Toyoda et al. the sensitivity was 60% and the specificity was 85% in HCC stage 1 (*n* = 235) when these three types of tumor markers were combined [74]. In Japan, the combination of these tumor markers is followed by a combination of imaging tests as a surveillance method for HCC. The GALAD score calculated from the age, sex, AFP, AFP-L3 fraction, and DCP has been reported to be useful in the early diagnosis of HCC globally [75] (Table 1). Moreover, PIVKA-II 1 mAU/mL = DCP 0.012 ng/mL can be calculated. In a study comparing NASH with HCC and without HCC at eight facilities in Germany, the GALAD score provided a better diagnosis of HCC (AUROC 0.93) than AFP (AUROC 0.88), AFP-L3 fractionation (AUROC 0.86), or PIVKA-II alone (AUROC 0.87) [76]. The GALAD score remained useful independent of the existence of liver cirrhosis; a cutoff value of 0.63 was appropriate, even though only 25 patients were examined using the Milan criteria. The sensitivity was good at 68%, the specificity at 95%, and an AUROC of 0.91. In a prospective study of 392 NAFLD patients (of which 17 experienced HCC incidence during the course) at Ogaki Municipal Hospital, the GALAD score was characterized by an upward trend from one and a half years before the diagnosis of HCC. The GALAD score was effective for the surveillance of NASH patients [76]. These are data from only a single facility; a multi-center validation study is needed in the future.

### 5.3. Apoptosis Inhibitor of Macrophages

The apoptosis inhibitor of macrophages (AIM) is a protein with a molecular weight of about 40 kD that was discovered by Professor Miyazaki of the University of Tokyo in 1999 [77]. AIM is produced by Kupffer cells in the liver and macrophages in the abdominal cavity [78]. IgM behaves as a carrier of the AIM protein, storing a large amount of the inactivated form of AIM in the blood. Under certain disease conditions, AIM can dissociate from IgM locally or systemically to exert its functions, inducing the removal of various biological debris, such as excess fat, bacteria, cancer cells, or dead cell debris [79]. In patients with NASH-HCC, AIM is more strongly dissociated from the IgM pentamer compared to non-tumor-bearing patients, while IgM-unbound AIM (free AIM) in blood increases in NASH-HCC [80]. Since free AIM (cutoff value: 1.6 μg/mL) can detect HCC with a higher sensitivity (88.5%) than PIVKA-II (53.8%) or AFP (26.9%), AIM is expected to be used as a diagnostic marker for detecting NASH-HCC. Since AIM may be used as a predictor of carcinogenesis in the future, researches should focus on future data collection. Since AIM has an inhibitory effect on HCC carcinogenesis in animal models [81,82], the clinical application of AIM for HCC treatment is also anticipated. The increased blood free AIM in NASH-HCC, moreover, may be a biodefense response.

### 5.4. SNP Combo

Various SNPs can relate to hepatocarcinogenesis in NAFLD. PNPLA3 SNP, which has the most abundant evidence, contributes not only to the development of hepatic fibrosis but also to hepatocarcinogenesis [20,83,84]. In a study from the United Kingdom, 100 Caucasian NAFLD associated HCC cases were reported. A study on 275 NAFLD non-carcinoma cases diagnosed by liver biopsy revealed that CG hetero carriers have a 2.52-fold higher risk, and GG homo carriers have a 12.19-fold higher risk of liver carcinogenesis than PNPLA3 CC homo carriers [83]. Since PNPLA3 GG homozygotes are a risk factor, even when examined in patients only with liver cirrhosis, PNPLA3 SNP GG homozygotes present a high risk of hepatocarcinogenesis independent of liver fibrosis. The cumulative hepatocarcinogenesis rate of 238 Japanese NAFLD patients diagnosed by biopsy was examined in PNPLA3 SNP [20]. GG homozygotes were shown to have significantly higher hepatocarcinogenesis rates than C allele carriers. A study on the risk of hepatocarcinogenesis among obese individuals using an obesity cohort in Sweden revealed that the G allele carriers had 5.9 (95% CI: 1.5–23.8) times higher hepatocarcinogenesis rates [24]. An analysis of the risk of hepatocarcinogenesis among confirmed Japanese diabetic patients revealed that the JAZF1 G allele and PNPLA3 SNP GG homozygotes are risk factors [85]. It has been reported that the T allele of membrane-bound O-acyl-transferase domain circulating 7 (MBOAT7) is involved in hepatocarcinogenesis in patients without cirrhosis [86]. We previously reported that a combination of PNPLA3 and dysferlin in patients with NAFLD in Japan presented a high risk of developing HCC for both risk alleles [87], but further cases need to be studied for validation. PNPLA3 G alleles are prevalent in Japan, South Korea, Taiwan, and Mexico [7]. Thus, there is concern that NASH-HCC incidence will increase in these countries. A report from Europe indicated that the risk alleles of PNPLA3, transmembrane 6 superfamily member 2 (TM6SF2) and hydroxysteroid 17-beta dehydrogenase 13 (HSD17B13), indicate 29 times the risk of hepatocarcinogenesis compared to the general population [67]. NAFLD patients with the TM6SF2 risk allele accumulate hepatic steatosis, but atherosclerosis is low in those with NAFLD risk alleles due to their excretion as very low density lipoprotein (VLDL). HSD17B13 modulates the action of the *PNPLA3* gene. Genotype of HSD17B13 are classified to allele homozygotes (T/T), heterozygotes (T/TA), and alternate allele homozygotes (TA/TA). When patients have the PNPLA3G allele with a TA variant of HSD17B13, inflammation and fibrosis are suppressed [88]. In this way, it is important to incorporate this SNP combo into risk assessment. However, this SNP poses problems for daily clinical practice, such as cost and the protection of personal information. It is important to use the family history of HCC and cirrhosis as a simple alternative method [89].

### 5.5. Noninvasive Liquid Biopsy

The concept of liquid biopsy was developed to address the need for reliable, minimally invasive methods for diagnosis, prognosis, and overall disease monitoring. Liquid biopsy is a modality where bodily fluid samples, instead of solid tissue, are used for pathophysiological or molecular analyses. This method has been introduced in many clinically relevant fields, including cancer research, and, in general, any bodily fluids can be used as potential samples for a liquid biopsy. The term “liquid biopsy” can also apply to cancer by-products, including circulating tumor cells (CTCs), cell-free DNA (cfDNA), cell-free RNA (cfRNA), microRNA (miRNA), extracellular vesicles (EVs), and tumor-derived metabolites [90]. The most widely used markers are CTCs and ctDNA. ctDNA that carries cancer-specific genetic and epigenetic aberrations may facilitate a noninvasive liquid biopsy for the diagnosis and monitoring of cancer [91,92].

## 6. Algorithm for HCC Surveillance in Nonalcoholic Fatty Liver Disease

**Figure 3:**
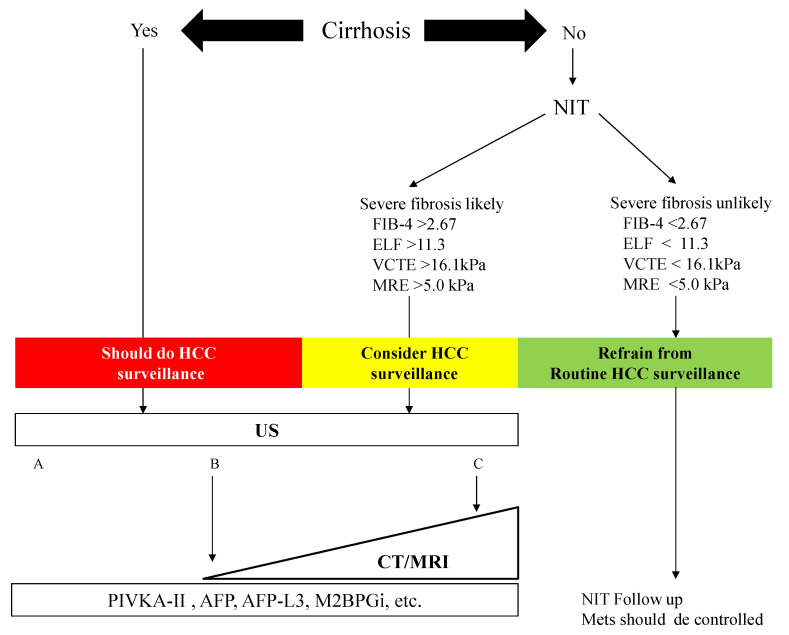
Algorithm for HCC surveillance in Nonalcoholic Fatty Liver Disease. NIT: noninvasive test, FIB-4: fibrosis-4, ELF: enhanced liver fibrosis, VCTE: vibration-controlled transient elastography, MRE: magnetic resonance elastography, HCC: hepatocellular carcinoma, US: ultrasonography, CT: computed tomography, MRI: magnetic resonance imaging, PIVKA-II: protein induced by vitamin K, AFP: α-fetoprotein, M2BPGi: Mac-2 binding protein glycosylated isomer. Mets: metabolic syndrome. The visualization score of the ultrasound for HCC screening is graded into the following categories: A—no or minimal limitations; B—moderate limitations, as the examination may obscure small masses; and C—severe limitation, as the examination may miss focal liver lesions [29].

We constructed an algorithm for HCC surveillance in NAFLD referring to the AGA expert review (Figure 3). NAFLD patients with cirrhosis should underdo HCC surveillance. NAFLD patients who are likely to have advanced fibrosis revealed by NITs (FIB-4 index, ELF test, VCTE, and MRE) should consider HCC surveillance. US is the first method for the surveillance of HCC, but the adequacy of US should be documented because of its difficulty to use among obese patients. In NAFLD patients with a high likelihood of an inadequate US (or if an US is attempted but inadequate), a CT or MRI may be utilized [29]. Tumor markers such as PIVKA-II, AFP, and AFP-L3 may help identify those with a high risk of HCC in NAFLD. NAFLD patients who are unlikely to have advanced fibrosis evaluated by NITs should not undergo routine surveillance.

## 7. Conclusions

The surveillance of HCC is unreasonable for all patients with NAFLD, who are estimated to total more than 2 billion worldwide. For cases of cirrhosis, suspected advanced fibrosis according to NITs, and cases of diabetes mellitus, HCC should be surveyed by semi-annual US and measurements of tumor markers such as PIVKA-II, AFP, and AFP-L3 (Figure 3). Because it is difficult to visualize the HCC in NAFLD patients by abdominal US because of obesity, alternative imaging such as CT or MRI should be considered. Early identification through surveillance provides more curative treatment options. If SNP measurements can be performed in general clinical settings, more efficient surveillance can be expected, but a cost-benefit analysis of this surveillance will be necessary in the future [93,94]. We also hope to establish innovative parameters for HCC surveillance, such as M2bpGi, the GALAD score, and free AIM. Precision tools that can better predict the development of HCC in individual patients with NAFLD are needed.

## Figures and Tables

**Table 1:** Noninvasive test (NIT) for stratifying a high risk of hepatocellular carcinoma (HCC) in nonalcoholic fatty liver disease (NAFLD).

NIT	Formula	HCC High Risk
FIB-4 index	(age [years] × AST [U/L]/(platelet count [10^9^/L] × √ALT [U/L]) https://www.eapharma.co.jp/medicalexpert/product/livact/fib-4/calculator.html	>2.67
NAFLD fibrosis score	−1.675 + 0.037 × age (years) + 0.094 × BMI (kg/m^2^) + 1.13 × impaired fasting glucose/diabetes (yes=1, no=0) + 0.99 × AST/ALT ratio − 0.013 × platelet count (×10^9^/L) - 0.66 × albumin (g/dL) http://nafldscore.com/	>0.676
ELF test	−7.412 + (In [HA] × 0.681) + (In [P3NP] × 0.775) + (In [TIMP1] × 0.494)	>11.3
GALAD score	10.08+1.67 × gender (male: 1, female:0) + 0.09 × age (years) + 2.34 × log10 (AFP [ng/mL]) + 0.04 × AFP-L3 (%) + 1.33 × log10 (DCP [ng/mL]) https://www.mdcalc.com/galad-model-hepatocellular-carcinoma-hcc	>−0.63

FIB-4: fibrosis-4, AST: aspartate aminotransferase, ALT: alanine aminotransferase, BMI: body mass index, HA: hyaluronic acid, P3NP: aminoterminal propeptide of type 3 procollagen. TIMP-1: tissue inhibitor of matrix metalloproteinase type 1, ELF: enhanced liver fibrosis, AFP: α fetoprotein, DCP: des-γ-carboxy pro-thrombin.

**Table 2:** Summary of the recommendations for HCC surveillance in nonalcoholic fatty liver disease [29].

Best Practice Advice 1	Screening for HCC should be considered in all patients with cirrhosis due to NAFLD
Best Practice Advice 2	Patients with NAFLD and noninvasive markers showing evidence of advanced liver fibrosis or cirrhosis should be considered for HCC screening
Best Practice Advice 3	Patients with NAFLD in the absence of advanced liver fibrosis should not be routinely considered for HCC screening
Best Practice Advice 4	The adequacy of ultrasound in assessing liver parenchyma for mass lesions should be documented when used for HCC screening in patients with cirrhosis due to NAFLD
Best Practice Advice 5	When the quality of ultrasonography is suboptimal for the screening of HCC (e.g., due to obesity), future screening should be performed by either computed tomography or a magnetic resonance imaging scan, with or without α-Fetoprotein, every 6 months
Best Practice Advice 6	Patients with cirrhosis due to NAFLD should be counseled on abstaining from alcohol drinking and tobacco smoking
Best Practice Advice 7	The optimal management of diabetes and dyslipidemia through lifestyle modifications and pharmacotherapy is encouraged for patients with NAFLD and advanced liver fibrosis who are at risk of HCC
Best Practice Advice 8	The optimal management of obesity through lifestyle modifications, pharmacotherapy, or endoscopic/surgical bariatric procedures is encouraged for patients with NAFLD and advanced liver fibrosis who are at risk of HCC

HCC: hepatocellular carcinoma, NAFLD: nonalcoholic fatty liver disease.

## Abbreviations

AASLD	American Association for the study of Liver Diseases
AFP	α-Fetoprotein
AFP-L3	Lens culinaris-agglutinin-reactive fraction of AFP
AGA	American Gastroenterology Association
AIM	Apoptosis inhibitor of macrophage
AST	aspartate aminotransferase
ALT	alanine aminotransferase
BMI	body mass index
HA	hyaluronic acid
P3NP	aminoterminal propeptide of type 3 procollagen
TIMP-1	tissue inhibitor of matrix metalloproteinase type 1
CTC	circulating tumor cells
CfDNA	cell-free DNA
cfRNA	cell-free RNA
EV	extracellular vesicle
DCP	des-γ-carboxy pro-thrombin
CI	confidence interval
CT	computed tomography
ELF	enhanced liver fibrosis
ELISA	enzyme linked immunosolvent assay
FIB-4	Fibrosis-4
NFS	NAFLD fibrosis score
HBV	hepatitis B virus
HCV	hepatitis C virus
HCC	hepatocellular carcinoma
HR	hazard ratio
HSD17B13	hydroxysteroid 17- beta dehydrogenase 13
FLD	fatty liver disease
M2BPGi	Mac-2 binding protein glycosylation isomer
MBOAT7	membrane bound O-acyl-transferase domain circulating 7
MDCT	multidetector CT
miRNA	microRNA
MRE	magnetic resonance elastography
MRI	magnetic resonance imaging
ECCM-MRI	extracellular contrast media-enhanced MRI
NAFLD	nonalcoholic fatty liver disease
NASH	nonalcoholic steatohepatitis
NIT	noninvasive test
TIMP-1	tissue inhibitor of matrix metalloproteinase type 1,
PIVKA-II	protein induced by vitamin K
P3NP	aminoterminal propeptide of type 3 procollagen
PNPLA3	patatin-like phospholipase domain-containing protein 3
QALY	quality-adjusted life year
RIA	radioimmunoassay
SNP	single nucleotide polymorphism
TIMP-1	tissue inhibitor of matrix metalloproteinase type 1
TM6SF	transmembrane 6 superfamily member 2
US	ultrasonography
VCTE	vibration-controlled transient elastography

## References

[1] Tateishi R., Uchino K., Fujiwara N., Takehara T., Okanoue T., Seike M., Yoshiji H., Yatsuhashi H., Shimizu M., Torimura T. (2019). A nationwide survey on non-B, non-C hepatocellular carcinoma in Japan: 2011–2015 update. J. Gastroenterol..

[2] Shim J.H. (2020). Should you advocate for hepatocellular carcinoma surveillance in patients with alcohol-related liver disease or non-alcoholic fatty liver disease?. Clin. Mol. Hepatol..

[3] Eslam M., Newsome P.N., Sarin S.K., Anstee Q.M., Targher G., Romero-Gomez M., Zelber-Sagi S., Wai-Sun Wong V., Dufour J.F., Schattenberg J.M. (2020). A new definition for metabolic dysfunction-associated fatty liver disease: An international expert consensus statement. J. Hepatol..

[4] Finelli C., Tarantino G. (2013). What is the role of adiponectin in obesity related non-alcoholic fatty liver disease?. World J. Gastroenterol..

[5] Younossi Z., Stepanova M., Ong J.P., Jacobson I.M., Bugianesi E., Duseja A., Eguchi Y., Wong V.W., Negro F., Yilmaz Y. (2019). Global Nonalcoholic Steatohepatitis Council. Nonalcoholic Steatohepatitis Is the Fastest Growing Cause of Hepatocellular Carcinoma in Liver Transplant Candidates. Clin. Gastroenterol. Hepatol..

[6] Younossi Z.M., Koenig A.B., Abdelatif D., Fazel Y., Henry L., Wymer M. (2016). Global epidemiology of nonalcoholic fatty liver disease-Meta-analytic assessment of prevalence, incidence, and outcomes. Hepatology.

[7] Younossi Z., Anstee Q.M., Marietti M., Hardy T., Henry L., Eslam M., George J., Bugianesi E. (2018). Global burden of NAFLD and NASH: Trends, predictions, risk factors and prevention. Nat. Rev. Gastroenterol. Hepatol..

[8] Williams C.D., Stengel J., Asike M.I., Torres D.M., Shaw J., Contreras M., Landt C.L., Harrison S.A. (2011). Prevalence of nonalcoholic fatty liver disease and nonalcoholic steatohepatitis among a largely middle-aged population utilizing ultrasound and liver biopsy: A prospective study. Gastroenterology.

[9] Yatsuji S., Hashimoto E., Tobari M., Taniai M., Tokushige K., Shiratori K. (2009). Clinical features and outcomes of cirrhosis due to non-alcoholic steatohepatitis compared with cirrhosis caused by chronic hepatitis C. J. Gastroenterol. Hepatol..

[10] Sumida Y., Yoneda M., Tokushige K., Kawanaka M., Fujii H., Yoneda M., Imajo K., Takahashi H., Eguchi Y., Ono M. (2020). Estimated Prevalence of Advanced Hepatic Fibrosis by Elastography in Patients with Type 2 Diabetes. Interv. Obes. Diabetes.

[11] Diehl A.M., Day C. (2017). Cause, Pathogenesis, and Treatment of Nonalcoholic Steatohepatitis. N. Engl. J. Med..

[12] Marrero J.A., Kulik L.M., Sirlin C.B., Zhu A.X., Finn R.S., Abecassis M.M., Roberts L.R., Heimbach J.K. (2018). Diagnosis, Staging, and Management of Hepatocellular Carcinoma: 2018 Practice Guidance by the American Association for the Study of Liver Diseases. Hepatology.

[13] Piscaglia F., Svegliati-Baroni G., Barchetti A. (2016). HCC-NAFLD Italian Study Group Clinical patterns of hepatocellular carcinoma in nonalcoholic fatty liver disease: A multicenter prospective study. Hepatology.

[14] Mittal S., Sada Y., El-Serag H.B., Kanwal F., Duan Z., Temple S., May S.B., Kramer J.R., Richardson P.A., Davila J.A. (2015). Temporal trends of nonalcoholic fatty liver disease-related hepatocellular carcinoma in the Veteran Affairs Population. Clin. Gastroenterol. Hepatol..

[15] Kanwal F., Kramer J.R., Mapakshi S., Natarajan Y., Chayanupatkul M., Richardson P.A., Li L., Desiderio R., Thrift A.P., Asch S.M. (2018). Risk of Hepatocellular Cancer in Patients with Non-Alcoholic Fatty Liver Disease. Gastroenterology.

[16] Ascha M.S., Hanouneh I.A., Lopez R., Tamimi T.A., Feldstein A.F., Zein N.N. (2010). The incidence and risk factors of hepatocellular carcinoma in patients with nonalcoholic steatohepatitis. Hepatology.

[17] Dam-Larsen S., Becker U., Franzmann M.B., Larsen K., Christoffersen P., Bendtsen F. (2009). Final results of a long-term, clinical follow-up in fatty liver patients. Scand. J. Gastroenterol..

[18] Soderberg C., Stal P., Askling J., Glaumann H., Lindberg G., Marmur J., Hultcrantz R. (2010). Decreased survival of subjects with elevated liver function tests during a 28-year follow-up. Hepatology.

[19] Kawamura Y., Arase Y., Ikeda K., Seko Y., Imai N., Hosaka T., Kobayashi M., Saitoh S., Sezaki H., Akuta N. (2012). Large-scale long-term follow-up study of Japanese patients with non-alcoholic Fatty liver disease for the onset of hepatocellular carcinoma. Am. J. Gastroenterol..

[20] Seko Y., Sumida Y., Tanaka S., Mori K., Taketani H., Ishiba H., Hara T., Okajima A., Umemura A., Nishikawa T. (2019). Development of hepatocellular carcinoma in Japanese patients with biopsy-proven non-alcoholic fatty liver disease: Association between PNPLA3 genotype and hepatocarcinogenesis/fibrosis progression. Hepatol. Res..

[21] White D.L., Kanwal F., El-Serag H.B. (2012). Association between nonalcoholic fatty liver disease and risk for hepatocellular cancer, based on systematic review. Clin. Gastroenterol. Hepatol..

[22] Leung C., Yeoh S.W., Patrick D., Ket S., Marion K., Gow P., Angus P.W. (2015). Characteristics of hepatocellular carcinoma in cirrhotic and non-cirrhotic non-alcoholic fatty liver disease. World J. Gastroenterol..

[23] Perumpail R.B., Wong R.J., Ahmed A., Harrison S.A. (2015). Hepatocellular carcinoma in the setting of non-cirrhotic nonalcoholic fatty liver disease and the metabolic syn-drome: US experience. Dig. Dis. Sci..

[24] Burza M.A., Pirazzi C., Maglio C., Sjöholm K., Mancina R.M., Svensson P.A., Jacobson P., Adiels M., Baroni M.G., Borén J. (2012). PNPLA3 I148M (rs738409) genetic variant is associated with hepatocellular carcinoma in obese individuals. Dig. Liver Dis..

[25] Dulai P.S., Singh S., Patel J., Soni M., Prokop L.J., Younossi Z., Sebastiani G., Ekstedt M., Hagstrom H., Nasr P. (2017). Increased risk of mortality by fibrosis stage in nonalcoholic fatty liver disease: Systematic review and meta-analysis. Hepatology.

[26] Plaz Torres M.C., Bodini G., Furnari M., Marabotto E., Zentilin P., Strazzabosco M., Giannini E.G. (2020). Surveillance for Hepatocellular Carcinoma in Patients with Non-Alcoholic Fatty Liver Disease: Universal or Selective?. Cancers.

[27] Yang J.D., Ahmed F., Mara K.C., Addissie B.D., Allen A.M., Gores G.J., Roberts L.R. (2020). Diabetes Is Associated With Increased Risk of Hepatocellular Carcinoma in Patients With Cirrhosis From Nonalcoholic Fatty Liver Disease. Hepatology.

[28] Taylor R.S., Taylor R.J., Bayliss S., Hagström H., Nasr P., Schattenberg J.M., Ishigami M., Toyoda H., Wai-Sun Wong V., Peleg N. (2020). Association Between Fibrosis Stage and Outcomes of Patients with Nonalcoholic Fatty Liver Disease: A Systematic Review and Meta-Analysis. Gastroenterology.

[29] Loomba R., Lim J.K., Patton H., El-Serag H.B. (2020). AGA Clinical Practice Update on Screening and Surveillance for Hepatocellular Carcinoma in Patients With Nonalcoholic Fatty Liver Disease: Expert Review. Gastroenterology.

[30] Chalasani N., Younossi Z., Lavine J.E., Charlton M., Cusi K., Rinella M., Harrison S.A., Brunt E.M., Sanyal A.J. (2018). The diagnosis and management of nonalcoholic fatty liver disease: Practice guidance from the American Association for the Study of Liver Diseases. Hepatology.

[31] Sumida Y., Shima T., Mitsumoto Y., Katayama T., Umemura A., Yamaguchi K., Itoh Y., Yoneda M., Okanoue T. (2020). Epidemiology, Pathogenesis, and Diagnostic Strategy of Diabetic Liver Disease in Japan. Int. J. Mol. Sci..

[32] Yoneda M., Imajo K., Takahashi H. (2018). Clinical strategy of diagnosing and following patients with nonalcoholic fatty liver disease based on invasive and noninvasive methods. J. Gastroenterol..

[33] Chan W.K., Treeprasertsuk S., Goh G.B., Fan J.G., Song M.J., Charatcharoenwitthaya P., Duseja A., Dan Y.Y., Imajo K., Nakajima A. (2019). Optimizing Use of Nonalcoholic Fatty Liver Disease Fibrosis Score, Fibrosis-4 Score, and Liver Stiffness Measurement to Identify Patients with Advanced Fibrosis. Clin. Gastroenterol. Hepatol..

[34] Castera L., Friedrich-Rust M., Loomba R. (2019). Noninvasive Assessment of Liver Disease in Patients with Nonalcoholic Fatty Liver Disease. Gastroenterology..

[35] Davyduke T., Tandon P., Al-Karaghouli M., Abraldes J.G., Ma M.M. (2019). Impact of Implementing a “FIB-4 First” Strategy on a Pathway for Patients with NAFLD Referred From Primary Care. Hepatol Commun..

[36] Sumida Y., Yoneda M., Hyogo H., Itoh Y., Ono M., Fujii H., Eguchi Y., Suzuki Y., Aoki N., Kanemasa K. (2012). Validation of the FIB4 index in a Japanese nonalcoholic fatty liver disease population. BMC Gastroenterol..

[37] Shah A.G., Lydecker A., Murray K., Tetri B.N., Contos M.J., Sanyal A.J., Nash Clinical Research Network (2009). Comparison of noninvasive markers of fibrosis in patients with nonalcoholic fatty liver disease. Clin. Gastroenterol. Hepatol..

[38] McPherson S., Hardy T., Dufour J.F., Petta S., Romero-Gomez M., Allison M., Oliveira C.P., Francque S., Van Gaal L., Schattenberg J. (2017). Age as a Confounding Factor for the Accurate Non-Invasive Diagnosis of Advanced NAFLD Fibrosis. Am. J. Gastroenterol..

[39] Ishiba H., Sumida Y., Tanaka S., Yoneda M., Hyogo H., Ono M., Fujii H., Eguchi Y., Suzuki Y., Yoneda M. (2018). The novel cutoff points for the FIB4 index categorized by age increase the diagnostic accuracy in NAFLD: A multi-center study. J. Gastroenterol..

[40] Ishiba Y., Sumida Y., Tanaka S., Yoneda M., Hyogo H., Ono M., Fujii H., Eguchi Y., Suzuki Y., Yoneda M. (2020). Type IV collagen 7S is the most accurate test for identifying advanced fibrosis in non-alcoholic fatty liver disease with type 2 diabetes. Hepatol. Commun..

[41] Srivastava A., Gailer R., Tanwar S., Trembling P., Parkes J., Rodger A., Suri D., Thorburn D., Sennett K., Morgan S. (2019). Prospective evaluation of a primary care referral pathway for patients with non-alcoholic fatty liver disease. J. Hepatol..

[42] Inadomi C., Takahashi H., Ogawa Y., Oeda S., Imajo K., Kubotsu Y., Tanaka K., Kessoku T., Okada M., Isoda H. (2020). Accuracy of the Enhanced Liver Fibrosis test, and combination of the Enhanced Liver Fibrosis and non-invasive tests for the diagnosis of advanced liver fibrosis in patients with non-alcoholic fatty liver disease. Hepatol. Res..

[43] Ogawa Y., Honda Y., Kessoku T., Tomeno W., Imajo K., Yoneda M., Kawanaka M., Kirikoshi H., Ono M., Taguri M. (2018). Wisteria floribunda agglutinin-positive Mac-2-binding protein and type 4 collagen 7S: Useful markers for the diagnosis of significant fibrosis in patients with non-alcoholic fatty liver disease. J. Gastroenterol. Hepatol..

[44] Okanoue T., Ebise H., Kai T., Mizuno M., Shima T., Ichihara J., Aoki M. (2018). A simple scoring system using type IV collagen 7s and aspartate aminotransferase for diagnosing nonalcoholic steatohepatitis and related fibrosis. J. Gastroenterol..

[45] Seko Y., Sumida Y., Tanaka S., Taketani H., Kanemasa K., Ishiba H., Okajima A., Nishimura T., Yamaguchi K., Moriguchi M. (2015). Predictors of malignancies and overall mortality in Japanese patients with biopsy-proven non-alcoholic fatty liver disease. Hepatol. Res..

[46] Yasui K., Hashimoto E., Komorizono Y., Koike K., Arii S., Imai Y., Shima T., Kanbara Y., Saibara T., Mori T. (2011). Characteristics of patients with nonalcoholic steatohepatitis who develop hepatocellular carcinoma. Clin. Gastroenterol. Hepatol..

[47] Ertle J., Dechene A., Sowa J.P., Penndorf V., Herzer K., Penndorf V., Herzer K., Kaiser G., Schlaak J.F., Gerken G. (2011). Non-alcoholic fatty liver disease progresses to hepatocellular carcinoma in the absence of apparent cirrhosis. Int. J. Cancer.

[48] Stine J.G., Wentworth B.J., Zimmet A., Rinella M.E., Loomba R., Caldwell S.H., Argo C.K. (2018). Systematic review with meta-analysis: Risk of hepatocellular carcinoma in non-alcoholic steatohepatitis without cirrhosis compared to other liver diseases. Aliment. Pharmacol. Ther..

[49] Harris P.S., Hansen R.M., Gray M.E., Massoud O.I., McGuire B.M., Shoreibah M.G. (2019). Hepatocellular carcinoma surveillance: An evidence-based approach. World J. Gastroenterol..

[50] Tobari M., Hashimoto E., Taniai M., Kodama K., Kogiso T., Tokushige K., Yamamoto M., Takayoshi N., Satoshi K., Tatsuo A. (2020). The characteristics and risk factors of hepatocellular carcinoma in nonalcoholic fatty liver disease without cirrhosis. J. Gastroenterol. Hepatol..

[51] Tokushige K., Hyogo H., Nakajima T., Ono M., Kawaguchi T., Honda K., Eguchi Y., Nozaki Y., Kawanaka M., Tanaka S. (2016). Hepatocellular carcinoma in Japanese patients with nonalcoholic fatty liver disease and alcoholic liver disease: Multicenter survey. J. Gastroenterol..

[52] Lin O.S., Keeffe E.B., Sanders G.D., Owens D.K. (2004). Cost-effectiveness of screening for hepatocellular carcinoma in patients with cirrhosis due to chronic hepatitis C. Aliment. Pharmacol. Ther..

[53] Arguedas M.R., Chen V.K., Eloubeidi M.A., Fallon M.B. (2003). Screening for hepatocellular carcinoma in patients with hepatitis C cirrhosis: A cost-utility analysis. Am. J. Gastroenterol..

[54] Nishie A., Goshima S., Haradome H., Hatano E., Imai Y., Kudo M., Matsuda M., Motosugi U., Saitoh S., Yoshimitsu K. (2017). Cost-effectiveness of EOB-MRI for Hepatocellular Carcinoma in Japan. Clin. Ther..

[55] He X., Wu J., Holtorf A.P., Rinde H., Xie S., Shen W., Hou J., Li X., Li Z., Lai J. (2018). Health economic assessment of Gd-EOB-DTPA MRI versus ECCM-MRI and multi-detector CT for diagnosis of hepatocellular carcinoma in China. PLoS ONE.

[56] Lee J.M., Kim M.J., Phongkitkarun S., Sobhonslidsuk A., Holtorf A.P., Rinde H., Bergmann K. (2016). Health economic evaluation of Gd-EOB-DTPA MRI vs ECCM-MRI and multi-detector computed tomography in patients with suspected hepatocellular carcinoma in Thailand and South Korea. J. Med. Econ..

[57] Singal A., Volk M.L., Waljee A., Salgia R., Higgins P., Rogers M.A., Marrero J.A. (2009). Meta-analysis: Surveillance with ultrasound for early-stage hepatocellular carcinoma in patients with cirrhosis. Aliment. Pharmacol. Ther..

[58] Trinchet J.C., Chaffaut C., Bourcier V., Degos F., Henrion J., Fontaine H., Roulot D., Mallat A., Hillaire S., Cales P. (2011). Ultrasonographic surveillance of hepatocellular carcinoma in cirrhosis: A randomized trial comparing 3- and 6-month periodicities. Hepatology.

[59] Kim G.A., Lee H.C., Choe J., Kim M.J., Lee M.J., Chang H.S., Bae I.Y., Kim H.K., An J., Shim J.H. (2018). Association between non-alcoholic fatty liver disease and cancer incidence rate. J. Hepatol..

[60] Kawaguchi T., Tokushige K., Hyogo H., Aikata H., Nakajima T., Ono M., Kawanaka M., Sawada K., Imajo K., Honda K. (2018). A Data Mining-based Prognostic Algorithm for NAFLD-related Hepatoma Patients: A Nationwide Study by the Japan Study Group of NAFLD. Sci. Rep..

[61] Tavakoli H., Robinson A., Liu B., Bhuket T., Younossi Z., Saab S., Ahmed A., Wong R.J. (2017). Cirrhosis Patients with Nonalcoholic Steatohepatitis Are Significantly Less Likely to Receive Surveillance for Hepatocellular Carcinoma. Dig. Dis. Sci..

[62] Younossi Z.M., Otgonsuren M., Henry L., Venkatesan C., Mishra A., Erario M., Hunt S. (2015). Association of nonalcoholic fatty liver disease (NAFLD) with hepatocellular carcinoma (HCC) in the United States from 2004 to 2009. Hepatology.

[63] Narimatsu H. (2015). Development of M2BPGi: A novel fibrosis serum glycobiomarker for chronic hepatitis/cirrhosis diagnostics. Expert Rev. Proteom..

[64] Alkhouri N., Johnson C., Adams L., Kitajima S., Tsuruno C., Colpitts T.L., Hatcho K., Lawitz E., Lopez R., Feldstein A.E. (2018). Serum Wisteria floribunda agglutinin-positive Mac-2-binding protein levels predict the presence of fibrotic nonalcoholic steatohepatitis (NASH) and NASH cirrhosis. PLoS ONE.

[65] Ito K., Murotani K., Nakade Y., Inoue T., Nakao H., Sumida Y., Kamada Y., Yoneda M. (2017). Serum Wisteria floribunda agglutinin-positive Mac-2-binding protein levels and liver fibrosis: A meta-analysis. J. Gastroenterol. Hepatol..

[66] Abe M., Miyake T., Kuno A., Imai Y., Sawai Y., Hino K., Hara Y., Hige S., Sakamoto M., Yamada G. (2015). Association between Wisteria floribunda agglutinin-positive Mac-2 binding protein and the fibrosis stage of non-alcoholic fatty liver disease. J. Gastroenterol..

[67] Tseng T.C., Peng C.Y., Hsu Y.C., Su T.H., Wang C.C., Liu C.J., Yang H.C., Yang W.T., Lin C.H., Yu M.L. (2020). Baseline Mac-2 Binding Protein Glycosylation Isomer Level Stratifies Risks of Hepatocellular Carcinoma in Chronic Hepatitis B Patients with Oral Antiviral Therapy. Liver Cancer.

[68] Kawaguchi K., Honda M., Ohta H., Terashima T., Shimakami T., Arai K., Yamashita T., Sakai Y., Yamashita T., Mizukoshi E. (2018). Serum Wisteria floribunda agglutinin-positive Mac-2 binding protein predicts hepatocellular carcinoma incidence and recurrence in nucleos(t)ide analogue therapy for chronic hepatitis B. J. Gastroenterol..

[69] Kim S.U., Heo J.Y., Kim B.K., Park J.Y., Kim D.Y., Han K.H., Park J.Y., Kim D.Y., Han K.H., Ahn S.H. (2017). Wisteria floribunda agglutinin-positive human Mac-2 binding protein predicts the risk of HBV-related liver cancer development. Liver Int..

[70] Shinkai N., Nojima M., Iio E., Matsunami K., Toyoda H., Murakami S., Inoue T., Ogawa S., Kumada T., Tanaka Y. (2018). High levels of serum Mac-2-binding protein glycosylation isomer (M2BPGi) predict the development of hepatocellular carcinoma in hepatitis B patients treated with nucleot(s)ide analogues. J. Gastroenterol..

[71] Cheung K.S., Seto W.K., Wong D.K., Mak L.Y., Lai C.L., Yuen M.F. (2017). Wisteria floribunda agglutinin-positive human Mac-2 binding protein predicts liver cancer development in chronic hepatitis B patients under antiviral treatment. Oncotarget.

[72] Mak L.Y., Ko M., To E., Wong D.K., Ma J.H., Hui T.L., Seto W.K., Fung J., Lai C.L., Yuen M.F. (2019). Serum Mac-2-binding protein glycosylation isomer and risk of hepatocellular carcinoma in entecavir-treated chronic hepatitis B patients. J. Gastroenterol. Hepatol..

[73] Kawanaka M., Tomiyama Y., Hyogo H., Koda M., Shima T., Tobita H., Hiramatsu A., Nishino K., Okamoto T., Sato S. (2018). Wisteria floribunda agglutinin-positive Mac-2 binding protein predicts the development of hepatocellular carcinoma in patients with non-alcoholic fatty liver disease. Hepatol. Res..

[74] Toyoda H., Kumada T., Tada T., Kaneoka Y., Maeda A., Kanke F., Satomura S. (2011). Clinical utility of highly sensitive Lens culinaris agglutinin-reactive alpha-fetoprotein in hepatocellular carcinoma patients with alpha-fetoprotein <20 ng/mL. Cancer Sci..

[75] Johnson P.J., Pirrie S.J., Cox T.F., Berhane S., Teng M., Palmer D., Morse J., Hull D., Patman G., Kagebayashi C. (2014). The detection of hepatocellular carcinoma using a prospectively developed and validated model based on serological biomarkers. Cancer Epidemiol. Biomark. Prev..

[76] Best J., Bechmann L.P., Sowa J.P., Sydor S., Dechêne A., Pflanz K., Bedreli S., Schotten C., Geier A., Berg T. (2020). GALAD Score Detects Early Hepatocellular Carcinoma in an International Cohort of Patients With Nonalcoholic Steatohepatitis. Clin. Gastroenterol. Hepatol..

[77] Miyazaki T., Hirokami Y., Matsuhashi N., Takatsuka H., Naito M. (1999). Increased susceptibility of thymocytes to apoptosis in mice lacking AIM, a novel murine macrophage-derived soluble factor belonging to the scavenger receptor cysteine-rich domain superfamily. J. Exp. Med..

[78] Arai S., Miyazaki T. (2014). Impacts of the apoptosis inhibitor of macrophage (AIM) on obesity-associated inflammatory diseases. Semin. Immunopathol..

[79] Miyazaki T., Yamazaki T., Sugisawa R., Gershwin M.E., Arai S. (2018). AIM associated with the IgM pentamer: Attackers on stand-by at aircraft carrier. Cell. Mol. Immunol..

[80] Koyama N., Yamazaki T., Kanetsuki Y., Hirota J., Asai T., Mitsumoto Y., Mizuno M., Shima T., Kanbara Y., Arai S. (2018). Activation of apoptosis inhibitor of macrophage is a sensitive diagnostic marker for NASH-associated hepatocellular carcinoma. J. Gastroenterol..

[81] Maehara N., Arai S., Mori M., Iwamura Y., Kurokawa J., Kai T., Kusunoki S., Taniguchi K., Ikeda K., Ohara O. (2014). Circulating AIM prevents hepatocellular carcinoma through complement activation. Cell Rep..

[82] Ozawa T., Maehara N., Kai T., Arai S., Miyazaki T. (2016). Dietary fructose-induced hepatocellular carcinoma development manifested in mice lacking apoptosis inhibitor of macrophage (AIM). Genes Cells..

[83] Liu Y.L., Patman G.L., Leathart J.B., Piguet A.C., Burt A.D., Dufour J.F., Day C.P., Daly A.K., Reeves H.L., Anstee Q.M. (2014). Carriage of the PNPLA3 rs738409 C >G polymorphism confers an increased risk of non-alcoholic fatty liver disease associated hepatocellular carcinoma. J. Hepatol..

[84] Seko Y., Yamaguchi K., Itoh Y. (2018). The genetic backgrounds in nonalcoholic fatty liver disease. Clin. J. Gastroenterol..

[85] Ueyama M., Nishida N., Korenaga M., Korenaga K., Kumagai E., Yanai H., Adachi H., Katsuyama H., Moriyama S., Hamasaki H. (2016). The impact of PNPLA3 and JAZF1 on hepatocellular carcinoma in non-viral hepatitis patients with type 2 diabetes mellitus. J. Gastroenterol..

[86] Donati B., Dongiovanni P., Romeo S., Meroni M., McCain M., Miele L., Petta S., Maier S., Rosso C., De Luca L. (2017). MBOAT7 rs641738 variant and hepatocellular carcinoma in non-cirrhotic individuals. Sci. Rep..

[87] Kawaguchi T., Shima T., Mizuno M., Mitsumoto Y., Umemura A., Kanbara Y., Tanaka S., Sumida Y., Yasui K., Takahashi M. (2018). Risk estimation model for nonalcoholic fatty liver disease in the Japanese using multiple genetic markers. PLoS ONE.

[88] Gellert-Kristensen H., Richardson T.G., Davey Smith G., Nordestgaard B.G., Tybjaerg-Hansen A., Stender S. (2020). Combined Effect of PNPLA3, TM6SF2, and HSD17B13 Variants on Risk of Cirrhosis and Hepatocellular Carcinoma in the General Population. Hepatology.

[89] Caussy C., Soni M., Cui J., Bettencourt R., Schork N., Chen C.H., Ikhwan M.A., Bassirian S., Cepin S., Gonzalez M.P. (2017). Familial NAFLD Cirrhosis Research Consortium. Nonalcoholic fatty liver disease with cirrhosis increases familial risk for advanced fibrosis. J. Clin. Investig..

[90] Mocan T., Simão A.L., Castro R.E., Rodrigues C., Słomka A., Wang B., Strassburg C., Wöhler A., Willms A.G., Kornek M. (2020). Liquid Biopsies in Hepatocellular Carcinoma: Are We Winning?. J. Clin. Med..

[91] Xu R.H., Wei W., Krawczyk M., Wang W., Luo H., Flagg K., Yi S., Shi W., Quan Q., Li K. (2017). Circulating tumor DNA methylation markers for diagnosis and prognosis of hepatocellular carcinoma. Nat. Mater..

[92] Li X., Wang H., Li T., Wang L., Wu X., Liu J., Xu Y., Wei W. (2020). Circulating tumor DNA/circulating tumor cells and the applicability in different causes induced hepatocellular carcinoma. Curr. Probl. Cancer.

[93] Srivastava A., Jong S., Gola A., Gailer R., Morgan S., Sennett K., Tanwar S., Pizzo E., O’Beirne J., Tsochatzis E. (2019). Cost-comparison analysis of FIB-4, ELF and fibroscan in community pathways for non-alcoholic fatty liver disease. BMC Gastroenterol..

[94] European Association for the Study of the Liver (EASL) European Association for the Study of Diabetes (EASD) European Association for the Study of Obesity (EASO) (2016). EASL-EASD-EASO Clinical Practice Guidelines for the management of non-alcoholic fatty liver disease. J. Hepatol..

